# Cost-effectiveness of excluding children with Shiga toxin-producing *Escherichia coli* (STEC) from childcare settings until microbiological clearance compared to return to childcare settings before microbiological clearance

**DOI:** 10.1017/S0950268825000196

**Published:** 2025-02-18

**Authors:** Dana Šumilo, Peter Auguste, Claire Jenkins, Jason Madan, Noel D. McCarthy

**Affiliations:** 1 Warwick Medical School, University of Warwick, Coventry, UK; 2 National Institute for Health and Care Research Health Protection Research Unit in Gastrointestinal Infections at University of Liverpool, Liverpool, UK; 3 UK Health Security Agency, London, UK; 4 Institute of Population Health, Trinity College Dublin, University of Dublin, Dublin, Ireland

**Keywords:** childcare, Children, cost-effectiveness, microbiological clearance, Shiga-Toxigenic *Escherichia coli*

## Abstract

Due to the risk of Shiga-toxin producing *Escherichia coli* (STEC) transmission, current guidance advises excluding young children from childcare settings until microbiologically clear. Children can shed STEC for a prolonged period, and the cost-effectiveness of exclusion has not been evaluated. Our decision tree analysis, including probabilistic sensitivity analysis, estimated comparative health system costs and effects of exclusion until microbiological clearance versus return to childcare setting before this. Due to the risk of secondary cases, return before microbiological clearance resulted in the incremental loss of 0.019 QALYs, but savings of £156. Using the willingness-to-pay threshold of £20000 per QALY, the incremental net monetary benefit of exclusion until microbiological clearance was £215. Exclusion until microbiological clearance remained cost-effective if the total costs for managing the exclusion were below £576. Return before microbiological clearance may, therefore, become cost-effective in cases where the costs of managing exclusion until microbiological clearance are high and/or the risk of secondary cases is very low. Broadening the decision perspective, including the costs of exclusion to the families, may also impact the recommendation. Further research is needed to assess the risk of STEC transmission from children who have clinically recovered and the impact of STEC and exclusion on families of the affected children.

## Background

Shiga-toxin producing *Escherichia coli* (STEC) are bacteria that can cause illness ranging from mild gastroenteritis to life-threatening haemolytic uraemic syndrome (HUS) and death. Children aged five years and under are at increased risk for person-to-person transmission of gastrointestinal infections including STEC and of severe illness following infection. The current UK Health Security Agency (UKHSA) operational guidance for STEC, therefore, recommends exclusion of young children with STEC O157, O26, and other higher risk strains from school, preschool, nursery, and similar childcare settings until microbiological clearance is evidenced by two negative consecutive stool samples once the child is symptom-free for 48 hours or more [1]. Other countries have similar guidelines [2].

The median duration of STEC shedding in children aged five years and under is 31 to 39 days, with between 8% to 18% of children shedding for more than two months [3, 4]. Fewer than 5% of children shed for more than three months [3, 4], but shedding for more than eight months has also been reported in literature [5]. When children experience shedding for a prolonged period, the guidance advises for the risk assessment to be reviewed to determine whether a supervised return to the childcare setting may be appropriate whilst awaiting microbiological clearance. There is a paucity of evidence comparing the public health benefit with costs from prolonged periods of exclusion to support the risk assessment for recovered and asymptomatic shedders [1].

The aim of this economic evaluation is, therefore, to estimate the cost-effectiveness of exclusion until microbiological clearance compared to return to childcare setting before microbiological clearance in children under the age of six years.

## Methods

To compare the impact of return and exclusion of children with STEC from childcare settings in the UK on costs and quality adjusted life years (QALYs) lost, a decision-analytic model was developed. QALYs are a summary measure combining the impact on quality and quantity of life associated with an intervention. One year of life in perfect health equals one QALY [6]. The model compared costs and QALYs lost due to excluding a child until microbiological clearance with costs and QALYs lost because of return to the childcare facility before microbiological clearance (due to the risk of transmission of STEC to other children). It was assumed that no QALYs were lost due to the child only shedding STEC, but having recovered clinically, and that parents would comply with the health protection advice to keep the child away therefore eliminating the risk of transmission in the childcare setting.

Table 1 describes the parameters that were used to populate the model and their data sources. The probability estimates for the risk of secondary STEC cases of different severity are based on published literature [4, 7–9]. As the risk of transmission from children who have clinically recovered from STEC has not been quantified [1], the estimate for secondary transmission is based on incidents in childcare facilities where an infectious child was in attendance and secondary transmission could be attributed to the childcare setting [4]. QALY loss estimates are based on previous literature, however, due to the lack of STEC-specific studies, some estimates are based on studies of other gastrointestinal diseases or chronic kidney disease arising from other causes using generic health-related Quality of Life (HRQoL) measures (EQ-5D and the Health Utilities Index HUI) and discounted for the value of life in the future years relative to the present. Healthcare costs associated with treatment of STEC are estimated using previously published estimates and the National Health Service (NHS) unit costs for 2019/2020 [10] and applying the discounting rate for the costs in the future.

**Table 1.** Model parameter descriptions and values.

Parameter	Description	Source estimate	Value
			**Proportion (95% CI)**
pSecondC	Probability of secondary transmission in a childcare setting due to return before microbiological clearance	Proportion of childcare facilities with secondary STEC cases where secondary transmission attributed to a childcare setting, Dabke et al (1)	0.036 (0–0.076)
SecondC	Number of symptomatic secondary cases in a childcare setting attended by an infectious case when transmission occurs	Ratio of symptomatic secondary versus primary cases attending a childcare setting, Dabke et al (1)	1 (1–2)*
pHospital	Probability of hospital admission among symptomatic secondary cases in children	Proportion of children with STEC in hospital, Adams et al (2)	0.40 (0.37–0.43)
pHUS	Probability of Haemolytic Uraemic Syndrome (HUS) in children hospitalized with STEC	Proportion of children hospitalized with STEC developing HUS, Adams et al (2)	0.48 (0.43–0.52)
pDeath	Probability of death in children with HUS	Proportion of children with HUS who died during hospitalization, Mody et al (3)	0.029 (0.017–0.040)
pSequalae	Probability of long-term sequelae in children with HUS	Rosales et al (4)	0.31 (0.25–0.37)
pSevereCKD−	Probability of severe chronic kidney disease (CKD) in children with long-term HUS sequelae	Proportion of children with HUS with long-term sequelae who had severe CKD, Buder et al (5)	0.21 (0.046–0.37)
			**Mean (95% CI)**
FollowUpDuration	Duration of follow-up until microbiological clearance (in weeks)	Mean duration of STEC shedding in children, Dabke et al (1)	4.14 (3.73–4.55)
QALYExclusion	QALYs lost due to exclusion until microbiological clearance	As the primary case has recovered physically during the period of exclusion until microbiological clearance, no QALYs would be lost due to the impact on physical health	0
QALYNoSecondC	QALYs lost if no secondary cases	No QALYs would be lost if there were no secondary cases	0
QALYNoHospital	QALYs lost due to secondary cases not resulting in hospital admission	Mean QALY loss due to gastroenteritis not requiring hospital admission, Marlow et al (6)	−0.0029 (–0.0034–0.0023)
QALYNoHUS	QALYs lost due to hospital admission for STEC, but no HUS	Mean QALY loss due to gastroenteritis requiring hospital admission, Marlow et al (6)	−0.0034 (–0.0053 to −0.0015)
QALYHUSRecovery	QALYs lost due to HUS, but full recovery	Mean QALY loss for one month for children on dialysis, Francis et al (7)	−0.033 (−0.041 to 0.024)
QALYMildCKD	Lifetime QALYs lost due to mild CKD	Mean lifetime QALY loss for children with mild CKD, Francis et al (7)	−6.08 (−7.15 to −5.01)
QALYSevereCKD	Lifetime QALYs lost due to severe CKD	Mean lifetime QALY loss for children with severe CKD, Francis et al (7)	−7.09 (−8.76 to −5.43)
QALYDeath	Discounted lifetime QALYs lost due to death	Discounted QALYs for children aged 0–9 years in United Kingdom, Briggs et al (8)	−25.33
AKIDialTime	Duration of dialysis for acute kidney injury (AKI)	Mean duration of dialysis in days for AKI in children who are likely to develop long term sequalae, Rosales et al (4)	14.65 (11.97–17.33)
			**Cost estimate in British pound sterling (±20%)**
cWeeklyFollowUpHPT	Weekly costs of Health Protection Team/ Environmental Health Officer time to following up cases until microbiological clearance	One hour weekly follow up time cost estimate	50 (40−60)
cLab	Laboratory testing costs for STEC	Estimated average O157 and non-O157 STEC laboratory testing costs	30 (24−36)
cReturnHPT	Health Protection Team/ Environmental Health Officer time for managing early return to childcare setting	Cost estimate for one hour of Health Protection Team/ Environmental Health Officer time	50 (40−60)
cDx	Costs of STEC diagnosis	Cost estimate for STEC diagnosis based on costs of an average two General Practitioner consultations (9) and two laboratory tests	138 (110.4−165.6)
cHPTSecondaryC	Costs for an incident meeting following secondary transmission	Cost estimates for four person hours	200 (160−240)
cOutbreakTest	Costs for testing close contacts if there is a secondary transmission	Cost estimate for ten laboratory tests	300 (240−360)
cHPTSevereOutbreak	Additional outbreak management costs if the secondary case developed HUS	Cost estimate of additional outbreak management costs	1000 (800 −1200)
cHospital	Costs for hospital stay with STEC, but no HUS	Non-elective short stay cost for paediatric, infectious or non-Infectious gastroenteritis, with clinical coding score 0 (10)	493 (394−592)
cAKI	Costs of AKI	Non-elective long stay cost for AKI without interventions, with clinical coding score 4–7 (10)	2,435 (1948−2922)
cICU	Paediatric critical care costs	Non-elective long stay Paediatric Intermediate Critical Care (10)	1,582 (1266−1898)
cAKIDial	Costs of renal dialysis for AKI	Peritoneal dialysis for AKI, 18 years and under (10)	113 (90−136)
cGPFollowup	Lifetime costs of additional General Practitioner consultations for patients with chronic kidney disease	Costs of two additional consultations per year (11)	2,085 (1668−2502)
cPaedNephr	Costs of paediatric nephrology appointments until adulthood	One paediatric nephrology outpatient appointment cost (10) per year until adulthood	4355 (3484 −5286)
cDial	Costs of dialysis prior to liver transplant	Two years of dialysis costs (11)	52731 (42185−63277)
cTranspl	Costs of kidney transplant	Kidney transplant for 18 years and under, from live donor (10)	22712 (18170−27254)
cNephr	Lifetime costs of nephrology appointments	One nephrology outpatient appointment cost (10) per year taking into account reduced life expectancy (12)	2057 (1645−2,468)
cOtherTreat	Lifetime costs of immunosuppression drugs	Costs of immunosuppression drugs (11) taking into account reduced life expectancy (12)	112884 (90307−135461)

* Mode (lowest and highest value)

1. Dabke G, Le Menach A, Black A, Gamblin J, Palmer M, Boxall N, et al. Duration of shedding of Verocytotoxin-producing Escherichia coli in children and risk of transmission in childcare facilities in England. Epidemiology & Infection. 2014;142 (2):327-34.

2. Adams N, Byrne L, Rose T, Adak B, Jenkins C, Charlett A, et al. Sociodemographic and clinical risk factors for paediatric typical haemolytic uraemic syndrome: retrospective cohort study. BMJ Paediatrics Open. 2019;3 (1):e000465.

3. Mody RK, Gu W, Griffin PM, Jones TF, Rounds J, Shiferaw B, et al. Postdiarrheal hemolytic uremic syndrome in United States children: clinical spectrum and predictors of in-hospital death. The Journal of Pediatrics. 2015;166 (4):1022-9.

4. Rosales A, Hofer J, Zimmerhackl L-B, Jungraithmayr TC, Riedl M, Giner T, et al. Need for long-term follow-up in enterohemorrhagic Escherichia coli-associated hemolytic uremic syndrome due to late-emerging sequelae. Clinical infectious diseases: an official publication of the Infectious Diseases Society of America. 2012;54 (10):1413-21.

5. Buder K, Werner H, Landolt MA, Neuhaus TJ, Laube GF, Sparta G. Health-related quality of life and mental health in parents of children with hemolytic uremic syndrome. Pediatric Nephrology. 2016;31 (6):923-32.

6. Marlow R, Finn A, Trotter C. Quality of life impacts from rotavirus gastroenteritis on children and their families in the UK. Vaccine. 2015;33 (39):5212-6.

7. Francis A, Didsbury MS, van Zwieten A, Chen K, James LJ, Kim S, et al. Quality of life of children and adolescents with chronic kidney disease: a cross-sectional study. Archives of Disease in Childhood. 2019;104 (2):134-40.

8. Briggs AH, Goldstein DA, Kirwin E, Meacock R, Pandya A, Vanness DJ, et al. Estimating (quality-adjusted) life-year losses associated with deaths: With application to COVID-19. Health Econ. 2021;30 (3):699-707.

9. Curtis LA, Burns A. Unit Costs of Health & Social Care 2020. PSSRU, University of Kent; 2020. p. 185.

10. NHS England. 2019/20 National Cost Collection Data Publication. 2021.

11. Kerr M. Chronic Kidney Disease in England: The Human and Financial Cost. Insight Health Economics; 2012.

12. Amaral S. Secular Trends in Survival Outcomes of Kidney Transplantation for Children: Is the Future Bright Enough? Clin J Am Soc Nephrol. 2020;15(3):308-10.

**Figure 1.**
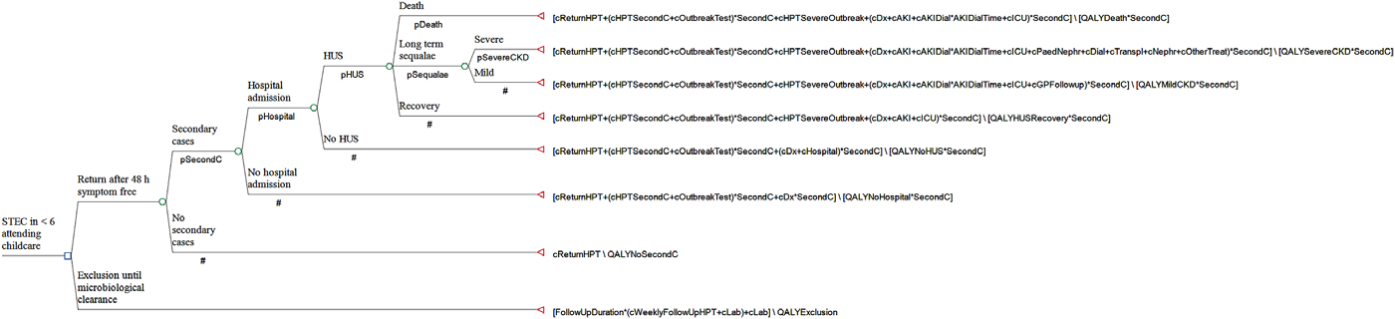
The decision tree comparing costs and outcomes of returning to childcare setting before microbiological clearance to exclusion until microbiological clearance.

**Figure 2.**
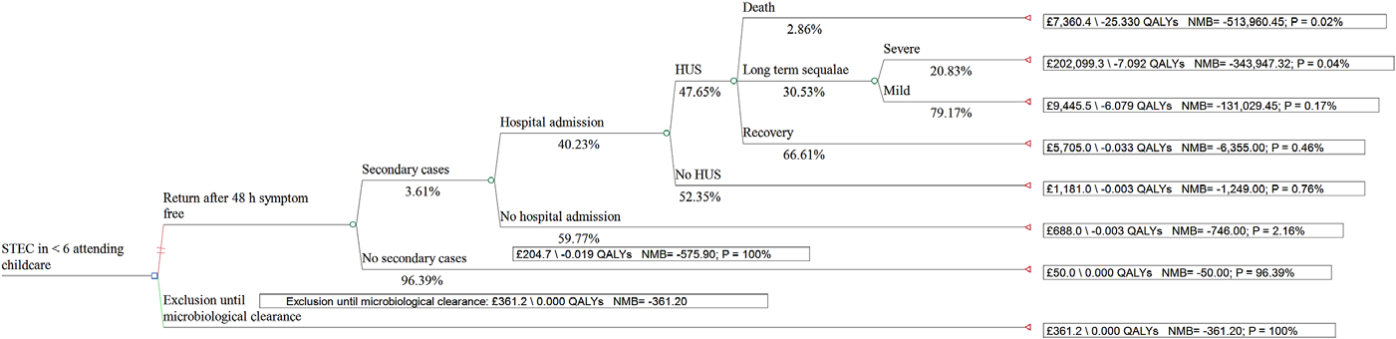
The results of the decision tree analysis.

The Decision Tree (Figure 1) compares the decision to return to the childcare setting before microbiological clearance to the child’s exclusion until microbiological clearance (routine practice which incurs health protection service costs due to the need to follow up the case and arrange regular laboratory testing until they have cleared the infection, and the second consecutive negative sample to confirm clearance [1]). If the child returns to the childcare facility before microbiological clearance, there is a risk of infecting other children with STEC. In a decision tree, at each stage, probabilities need to add up to 100% as they represent all the possible outcomes that could happen at that point. The overall probability of a specific outcome is calculated by multiplying all the probabilities along the relevant path. The values of the parameters detailed in Table 1 allowed for estimating the probabilities for each part of the decision tree. For example, the estimated probability of having a symptomatic secondary STEC infection not requiring hospital admission was 2.2% (pSecondC x (1-pHospital) x100). The probability of a symptomatic secondary STEC infection requiring hospital admission was 1.5%, the probability of developing HUS was 0.69%, the probability of developing long-term sequelae following HUS was 0.21%, the probability of severe long-term sequelae was 0.044% and the probability of death was 0.020%. Figure 2 summarises probability estimates for each of the final outcomes considered in the analysis.

As an early return needs to be arranged with the childcare setting, this incurs costs due to health protection service time, but if there are no secondary cases, no other costs would be incurred. If there is secondary transmission following an early return to the childcare facility, there will be additional costs to the health protection service, including contact testing and incident management costs, with higher costs if a secondary case develops severe infection. There also will be costs to the health service for treating secondary cases which depend on severity. In addition, there will be QALY loss for any secondary case, which will be the lowest for cases experiencing mild infection and the highest in the case of death.

The cost-effectiveness analysis was undertaken according to best practices for conducting and reporting economic analyses. Our base case analysis took the perspective of the healthcare system and did not include costs and welfare loss to the families caused by STEC and exclusion from childcare. Incremental Net Monetary Benefit (NMB) analysis was undertaken using the NICE willingness-to-pay threshold of £20,000 per QALY [6]. We performed a one-way sensitivity analysis using the values of the model parameters in Table 1 to assess which of the parameters have the greatest impact on the results. To take account of the joint uncertainty in the parameter estimates used in the model, we also undertook probabilistic sensitivity analysis. We applied a beta distribution for all probability estimates, triangular distribution for the number of secondary cases (SecondC), and normal distribution for all other parameters, except for costs for which we used the mean estimates detailed in Table 1. Monte Carlo simulation was used to sample from the distribution of each of these parameters, running 10000 replications of the model, to estimate the costs and QALYs lost for each strategy. We also undertook a threshold analysis to determine how our recommendations might change were we to broaden our perspective to include additional costs and benefits such as costs and welfare loss to the families caused by STEC and exclusion from childcare. All modelling was conducted using TreeAge Pro Healthcare 2022 version R2.1 [11].

### Patient, Public, and Stakeholder Involvement

This work is a partnership with the clinical stakeholders at the UK Health Security Agency (UKHSA), as part of the Nation Institute for Health and Care Research (NIHR) Health Protection Research Unit in Gastrointestinal Infections (GI) and is undertaken to help inform risk assessments and the UKHSA’s public health operational guidance for STEC. We also discussed our STEC research with six public contributors (including parents and people who have worked with and managed childcare settings for nursery-aged children). They stressed the importance of balancing the impact on the excluded child and their family (with the impact varying depending on the availability of financial resources, social networks, and educational support at home) and the risk of onward transmission to other children (with the gap in evidence regarding the likelihood of transmission at different stages of STEC infection). They also acknowledged the difficulty of estimating the costs and welfare impact as there are very many different factors at play that would affect them. Public contributors also suggested alternative wording, such as ‘asked to stay away’ and ‘not to come into a childcare setting’ instead of ‘exclusion’ when communicating about this work with the non-specialist audience and stressed the need to clearly explain to parents the risks associated with STEC onward transmission.

## Results

Exclusion until microbiological clearance resulted in estimated costs of £361 due to the need to follow up the case to arrange testing of faecal samples until the acquisition of two consecutive negative specimens, but no loss of QALYs as the child would have already recovered from clinical illness during this period (Figure 2). The estimated costs of the alternative strategy allowing early return to the childcare setting after clinical recovery resulted in estimated costs of £205 and loss of 0.019 QALYs. The costs for this strategy ranged from health protection service costs of £50 if early return to the childcare facility did not result in secondary transmission and no loss of QALYs, to £19,148 due to health protection and healthcare costs if secondary transmission led to another child developing HUS with associated loss of 2.7 QALYs. Costs and QALY loss were particularly high if the secondary case developed severe long-term sequelae following HUS or died (Figure 2).

**Figure 3.**
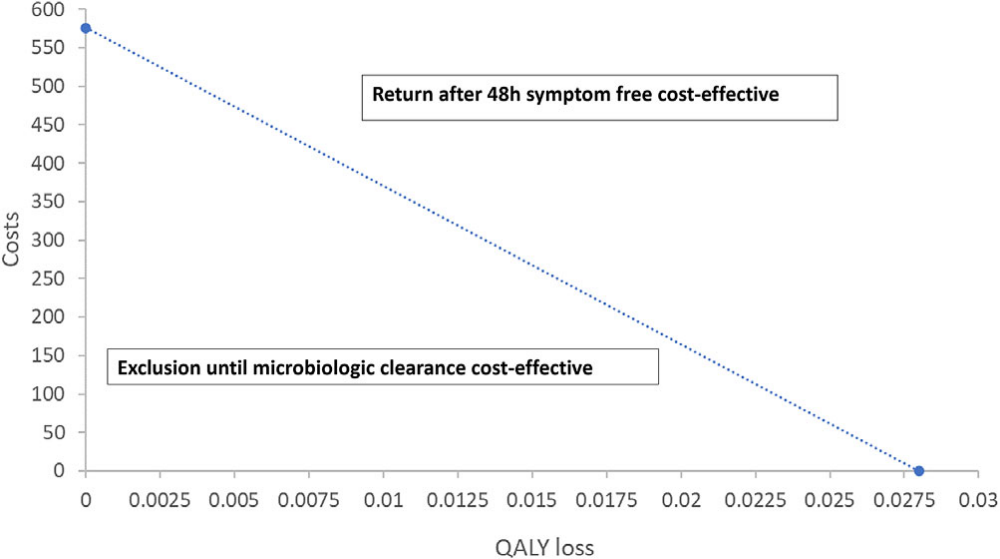
Results of threshold analysis using the willingness-to-pay threshold of £20000 per QALY.

The model shows that, due to the risk of secondary cases, return to childcare setting before microbiological clearance resulted in an incremental gain of £156, but the loss of 0.019 QALYs compared to exclusion until microbiological clearance. The incremental NMB of exclusion until microbiological clearance, applying the willingness-to-pay threshold of £20,000 per QALY, was £215. Threshold analysis demonstrates that exclusion until microbiological clearance is cost-effective if additional costs (such as costs of exclusion due to productivity loss) do not increase the total costs above £576, if the health and welfare loss, including to the family of the excluded child was below 0.0275, or any linear combination of the two (Figure 3).

**Figure 4.**
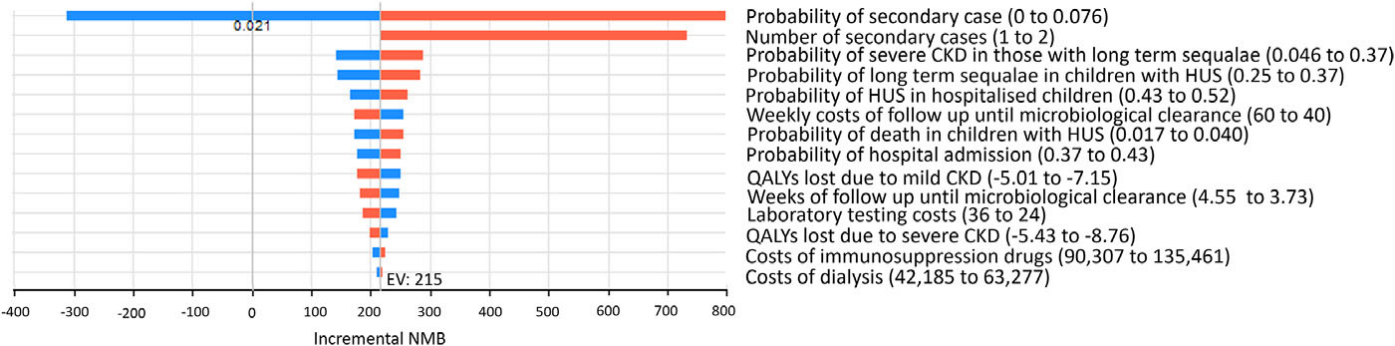
Tornado diagram for the incremental NMB of exclusion until microbiological clearance vs early return (after 48 h symptom free) before microbiological clearance.

Results of the one-way sensitivity analysis in Figure 4 show that the probability of having a secondary case has the greatest influence on the results with exclusion until microbiological clearance ceases to be the more cost-effective strategy if the probability of secondary transmission in the childcare setting is below 2.1%.

**Figure 5.**
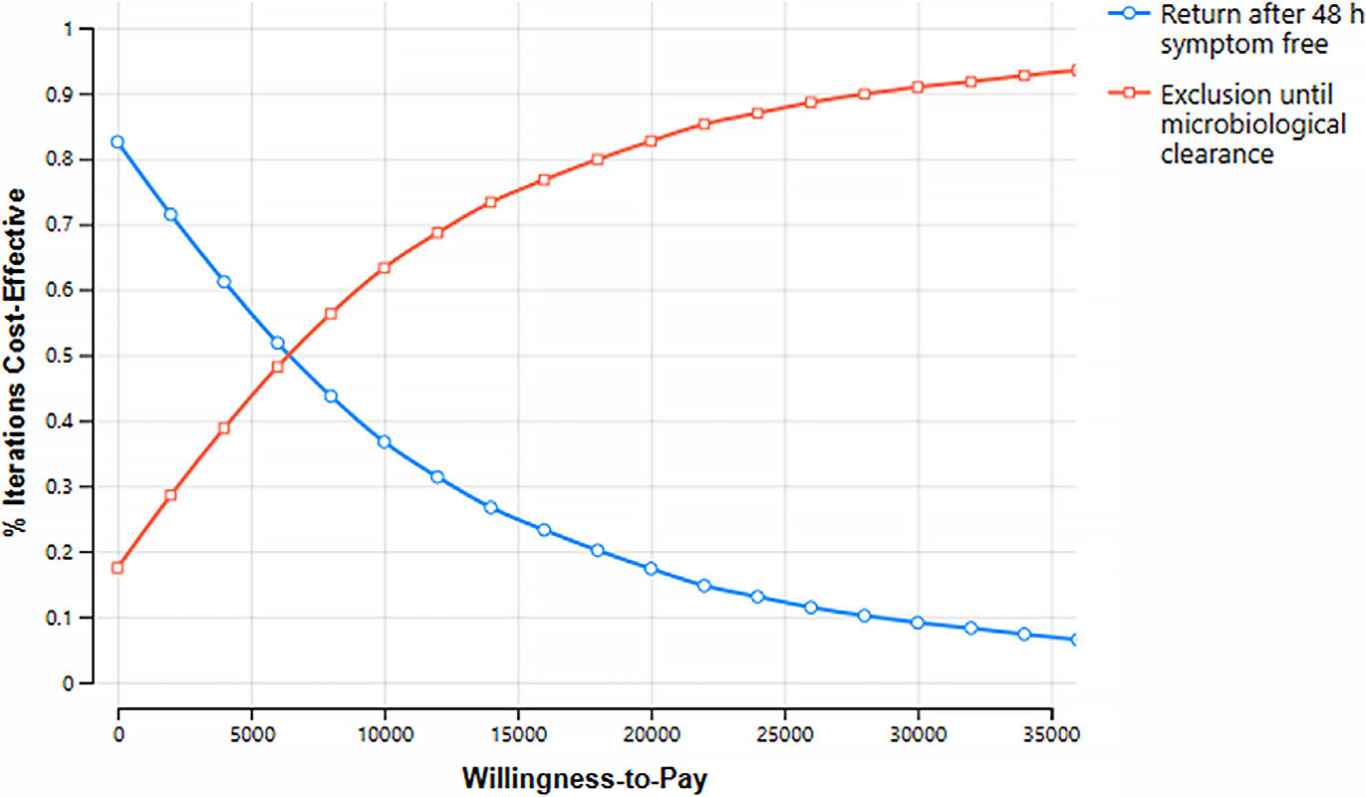
Cost-Effectiveness Acceptability Curve (CEAC) illustrating probability of cost-effectiveness for each strategy across a range of QALY willingness-to-pay values.

At a willingness-to-pay threshold of £20000 per QALY, results of probabilistic sensitivity analysis showed that exclusion until microbiological clearance was the optimal strategy in the majority (83%) of model calculations (Figure 5).

## Discussion

Our results suggest that the current practice of excluding children from childcare settings until microbiological clearance is cost-effective from a healthcare perspective, for the average duration of STEC follow-up until microbiological clearance. However, our sensitivity analyses suggest this strategy may cease to be cost-effective if the risk of secondary transmission to other children in the childcare setting is very low or if there are additional costs and wellbeing losses to the families of excluded children. Difficulties that health protection teams face in implementing exclusion until microbiological clearance have been documented, due to exclusion causing disruption to family, including financial and childcare issues [4].

Our analysis followed standard practice for economic evaluation of individual-level health care interventions and did not include costs and the impact on QoL of families of children with STEC as the result of secondary transmission or the costs and the impact on the child or the family of the child excluded from childcare until microbiological clearance. These costs and impact on the families can vary, but there is a paucity of studies investigating the experiences of these families and the impact of exclusion of young children from childcare settings due to STEC infection and how it differs based on family circumstances and duration of exclusion. Our public involvement contributors highlighted the potential importance of this wider perspective for a public health intervention restricting access to childcare and usual social activities. If a wider societal perspective were taken considering these costs and who would pay them the most cost-effective strategy might change. Our threshold analysis illustrates the magnitude of additional costs and benefits that would need to be assumed for exclusion till microbiological clearance to no longer cost-effective. In any scenario where the combination of health protection management costs and costs borne by the household exceeds £576, the cost-effectiveness would be lost. This is very plausible with a loss of income. However, balancing social and financial costs for avoided exclusion among families into which transmission is prevented should also then be considered. These are complex and important issues for a fuller application of cost-effectiveness analysis to public health interventions such as these.

This health economic evaluation has several limitations. Firstly, although asymptomatic children can transmit STEC, the risk of transmission is difficult to quantify [1] and we relied on estimates of the risk of transmission from children while infectious usually when symptomatic at the beginning of the illness with the median of two days of attendance before exclusion [4]. Although the infective dose for STEC is thought to be very low [12], the risk of secondary transmission may be different from asymptomatic clinically recovered shedders, and could also be impacted by a longer duration of exposure after supervised return to the childcare facility. Additionally, our estimates are primarily based on the most common STEC O157, but the risk of transmission and severity of the disease may vary by STEC serotype and virulence profile, as well as other factors, such as child’s age, hygiene standards, and childcare facility [1]. Our analysis also assumes that by asking parents of the infected child to stay away from the childcare setting until microbiological clearance, the risk of transmission in the childcare setting is eliminated and that parents will comply with this health protection advice. Furthermore, there is also a paucity of studies assessing the costs and impact on QoL for children and their families depending on the severity of STEC infection. Where previous QoL studies exist, they could not always be mapped to the generic health status measure EQ-5D.

In summary, this is the first economic evaluation of current exclusion guidance in England. Our findings suggest that while exclusion until microbiological clearance is cost-effective from a healthcare perspective alone, supervised return to childcare setting before microbiological clearance may become cost-effective in circumstances where the likelihood of secondary cases is low, and the costs of exclusion are high, for example, due to productivity and wellbeing losses to the family due to prolonged shedding of STEC in a clinically recovered child. There are, nevertheless, ethical considerations related to returning to childcare before microbiological clearance as parents of other children attending the childcare facility would have to accept a small risk of their child also acquiring an STEC infection. Further research is needed to quantify the risk of secondary transmission from clinically recovered children with STEC infection, STEC-specific cost and QALY estimates, and parental preferences with regard to the risks and benefits to allow for refinement of the cost-effectiveness model. Including lost productivity and wellbeing may change the recommendation, but further research is needed to quantify these impacts. There is also a need to assess the cost-effectiveness of other pathogens requiring exclusion until microbiological clearance [13].

## Data Availability

This study used data from previously published studies and publicly available data sources.
